# The BAH domain of BAHD1 is a histone H3K27me3 reader

**DOI:** 10.1007/s13238-016-0243-z

**Published:** 2016-02-05

**Authors:** Dan Zhao, Xiaojie Zhang, Haipeng Guan, Xiaozhe Xiong, Xiaomeng Shi, Haiteng Deng, Haitao Li

**Affiliations:** College of Life Sciences, Peking University, Beijing, 100871 China; MOE Key Laboratory of Protein Sciences, Beijing Advanced Innovation Center for Structural Biology, Department of Basic Medical Sciences, School of Medicine, Tsinghua University, Beijing, 100084 China; Center for Biomedical Analysis, School of Medicine, Tsinghua University, Beijing, 100084 China; Collaborative Innovation Center for Biotherapy, West China Hospital, Sichuan University, Chengdu, 610041 China

**Dear Editor,**

Histone recognition by reader modules constitutes a major mechanism for epigenetic regulation (Jenuwein and Allis 2001). BAHD1 (bromo adjacent homology domain containing protein 1) is a vertebrate-specific nuclear protein (Fig. S1) involved in gene silencing by promoting heterochromatin formation. BAHD1 is characteristic with an N-terminal proline-rich region, a nuclear localization signal motif, and a C-terminal bromo adjacent homology (BAH) domain (Fig. 1A). Previous study revealed that BAHD1 could act as a scaffold protein and tether diverse heterochromatin-associated factors including HP1, MBD1, SETDB1, HDAC5, and several transcriptional factors to trigger facultative heterochromatin formation (Bierne, Tham et al. 2009). Consistent with a “repressive” role, BAHD1 binds to CpG-rich P3 promoter region of *IGF2* (insulin-like growth factor II) then represses *IGF2* and *IGF2* antisense transcription via the recruitment of MBD1 and HDAC5 (Bierne, Tham et al. 2009). Intriguingly, BAHD1 is also involved in host-pathogen interplay. For example, at early *L. monocytogenes* infection state, BAHD1 forms a complex with TRIM28 and HP1 to repress interferon-stimulated genes, including IFNL1, IFNL2, and IFNL3 . At specific infection stages, *Listeria* secretes a virulence factor, LntA, which could physically interact with BAHD1 to activate interferon (IFN)-stimulated genes (ISGs) (Lebreton, Lakisic et al. 2011). Despite a repressive role of BAHD1, the molecular mechanism underlying BAHD1 heterochromatin targeting remains largely unexplored.

**Figure 1:**
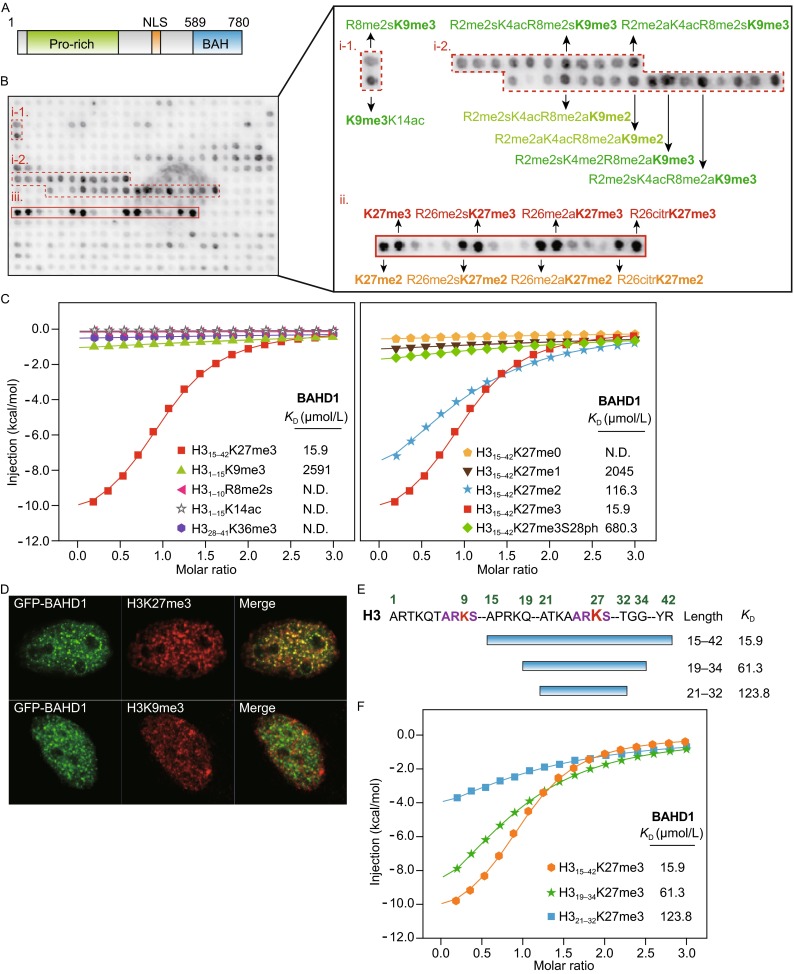
**The BAHD1 BAH is an H3K27me3-binding domain**. (A) Domain architecture of human BAHD1 protein. (B) A modified histone peptide array screen probed with GST-tagged BAH_BAHD1_ domain. Spots were detected by anti-GST antibody. Positive hits were labeled with red boxes and the corresponding peptides information was annotated on the right. i-1 & i-2, peptides that contain K9me2/me3; ii, peptides that contain K27me2/me3. (C) Isothermal titration calorimetry (ITC) fitting curves of BAH_BAHD1_ domain with indicated histone peptides. N.D., not detected. Peptide sequences and complete thermodynamic parameters were listed in Table S1. (D) Immunofluorescence of EGFP-BAHD1 (full length) transfected HeLa cells labeled with H3K27me3 and H3K9me3 antibodies. (E) ITC fitting curves of BAH_BAHD1_ domain titrated with different frames of the H3K27me3 peptides. (F) Diagram and sequence information of histone H3K27me3 peptides used in the ITC titration

BAH domain is an evolutionarily conserved motif which is found in several chromatin-associated proteins such as Sir3, ORC1, Rsc2, ZMET2, and DNMT1. BAH is characteristic of a conserved β-sheet core (typically 9-bladed) flanked with function-specific N-, C-, and β6-β7 insertions. Recent structural and functional studies revealed multi-facet roles of BAH domain in chromatin regulation (Yang and Xu 2013). For example, in yeast, ORC1 BAH could act as a scaffold to mediate ORC1-Sir1 interaction to form a silencing complex; Sir3 BAH could function as a nucleosome-targeting unit to induce heterochromatin formation; moreover, Rsc2 BAH could bind histone H3 and its interaction interface is conserved in a subset of Rsc-like BAH domains (Chambers, Pearl et al. 2013). Interestingly, in metazoan species, ORC1 BAH domain has acquired a histone methylation reader activity and recognizes H4K20me2 to prompt DNA replication licensing (Kuo, Song et al. 2012). BAH domains also exist in DNA methyltransferases of mammalian DNMT1 and plant ZMET2. Noteworthily, ZMET2 but not DNMT1 BAH domain displays histone H3K9me2 binding activity and thus directly mediates a cross-talk between histone and DNA methylations (Du, Johnson et al. 2015). Previous study showed that deletion of the C-terminal BAH domain interfered with co-localization of BAHD1 with H3K27me3 at nuclear foci *in vivo* (Bierne, Tham et al. 2009), suggesting a role of BAH_BAHD1_ in histone H3K27me3 recognition. In order to test this hypothesis, we recombinantly expressed BAH_BAHD1_ (aa 589–780) with an N-terminal GST tag, and carried out modified histone peptide array screening (Fig. 1B). Many black dots of different intensities were detected in the grid, supporting a histone binding activity of BAH_BAHD1_. In-depth data analysis revealed that all the positive hits can be classified into two major categories: the H3K9me2/3-containing and the H3K27me2/3-containing clusters. Among these hits, H3K27me3-containing peptides displayed strongest signal, consistent with a proposed role of BAH_BAHD1_ in H3K27me3 recognition.

In order to quantitatively characterize the array data, we performed isothermal titration calorimetry (ITC) under an optimized buffer condition that gives a better melting temperature (Tm_optimized_ = 39°C vs. Tm_unoptimized_ = 35.5°C) of BAH_BAHD1_ in thermal shift assays (TSA) (Fig. S2). ITC titration revealed a dissociation constant (*K*_D_) of 15.9 μmol/L between BAH_BAHD1_ and H3_15-42_K27me3 peptide. By contrast, the binding affinity dropped to 2.6 mmol/L in the case of H3_1-15_K9me3, and no bindings were observed for H3_1-10_R8me2s, H3_1-15_K14ac, and H3_28-41_K36me3 peptides (Fig. 1C, left), suggesting K27 site-specificity. We next explored the methylation-state preference of H3K27 by BAH_BAHD1_. Following the loss of methyl groups, the binding affinity dropped 8-fold for H3K27me2 (*K*_D_ = 116 μmol/L), 129-fold for H3K27me1 (*K*_D_ = 2.0 mmol/L) and lost completely for unmodified H3K27, suggesting BAH_BAHD1_ is a histone H3K27 trimethyllysine-specific reader (Fig. 1C, right). Remarkably, phosphorylation of H3S28 (H3S28ph) dramatically reduced H3K27me3 binding by 42-fold (*K*_D_ = 680 μmol/L), suggesting a “methyl-phos” binary switch mechanism of BAH_BAHD1_ (Fischle, Wang et al. 2003).

Both H3K9me3 and H3K27me3 are hallmarks for heterochromatin and gene silencing (Kim and Kim 2012). To evaluate the *in vivo* functional distinction between the two marks in BAHD1 recruitment, we next performed co-localization analysis of BAHD1 with H3K9me3 or H3K27me3 in HeLa cells by immunofluorescence. As shown in Fig. 1D, ectopically expressed EGFP-BAHD1 overlapped nicely with the punctate staining pattern of H3K27me3 as evidenced by the yellow appearance of merged signals. By contrast, little overlapping between EGFP-BAHD1 and H3K9me3 was observed. This result confirms the functional connection of BAHD1 with H3K27me3 but not H3K9me3 at cellular level. H3K9me3 and H3K27me3 share a common “ARKS” sequence motif (Fig. 1E). Additional ITC titrations using H3K27me3 peptides in shorter H3 frames of 19–34 and 21–32 revealed 3.9- and 7.8-fold binding reduction (Fig. 1F), suggesting that distal sequence motifs other than “ARKS” contribute to H3K27me3 recognition and thus discriminate against H3K9me3.

We next performed hydrogen exchange mass spectrometry (HXMS) to map the responsible regions of BAH_BAHD1_ for histone H3_15-42_K27me3 peptide binding (Wales and Engen 2006). Three peptide segments spanning “633–653”, “666–691”, and “674–698” of BAHD1 displayed obvious reduced exchange rates in the presence of the H3K27me3 peptide (Fig. 2A), suggesting their involvement in histone recognition. By contrast, other peptides show little or no change in deuterium uptake level upon binding to histone ligand. Structural modelling of BAH_BAHD1_ revealed that the three peptide segments cluster together to form a surface that contains an aromatic cage for methyllysine binding (Figs. 2B and S3). This aromatic cage is formed by Y645, W667, and Y669, which are conserved among the BAH domains of mouse ORC1 and plant ZMET2 that are known to recognize H4K20me2 and H3K9me2, respectively (Fig. S4).

**Figure 2:**
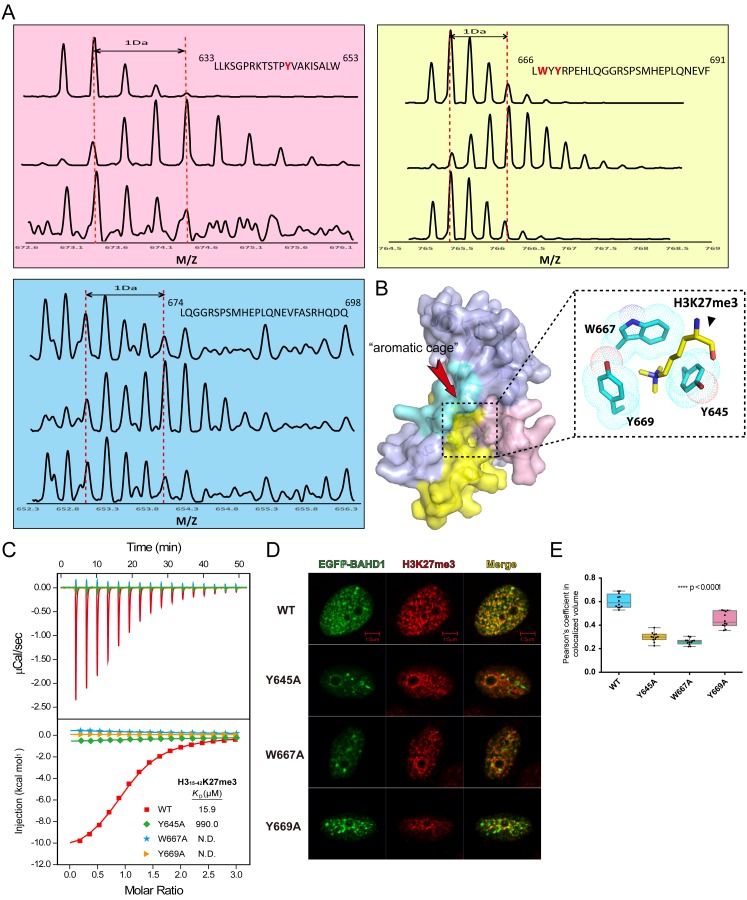
**An aromatic cage is required for H3K27me3 recognition by BAH**_**BAHD1**_ **domain**. (A) Hydrogen exchange MS (HXMS) analysis of BAH_BAHD1_-H3K27me3 complexion. Representative deuterium exchange mass spectra of BAH_BAHD1_ peptide fragments. Sections of BAH_BAHD1_ peptides from 633–653 are shown in pink; 666–691 in yellow and 674–698 in blue (In each section, top: undeuterated sample; middle: BAH_BAHD1_ only peptides after 10 min in deuterated buffer; down: BAH_BAHD1_ with H3K27me3 after 10 min in deuterated buffer). (B) H3K27me3 binding region mapping of BAH_BAHD1_ based on the HX MS data. The structure model of BAH_BAHD1_ was obtained by homologous modelling. BAH_BAHD1_ structure is shown in surface view with the corresponding H3K27me3 binding region color coded pink, yellow, and cyan corresponding to panel (A). Arrow highlights the aromatic cage formed at the center of BAH_BAHD1_. A close-up view of the aromatic cage with a modelled H3K27me3 ligand is shown top-right. (C) ITC fitting curves of histone H3_15-42_K27me3 peptide with wild type (WT) and mutant BAH _BAHD1_ (Y645A, W667A, and Y669A). (D) Immunofluorescence of EGFP-BAHD1 and mutants transfected HeLa cells labeled with H3K27me3 antibody. Scale bar represents 5 μm. (E) Quantification of EGFP-BAHD1 and mutants signals co-localized with H3K27me3. Counts are based on 12 interphase cells in individual clones and the Pearson’s coefficient of each cell was listed in Table S2

To test the importance of the aromatic residues in H3K27me3 readout by BAH_BAHD1_, we generated single point mutant of Y645A, W667A, Y669A, and performed ITC titration using H3_15-42_K27me3 peptide. As expected, alanine mutation of the aromatic residues disrupted binding between BAH_BAHD1_ and H3K27me3 peptide (Fig. 2C), supporting a critical role of the aromatic cage in methyllysine recognition. The importance of these aromatic residues was further confirmed by immunofluorescence analysis in HeLa cells. As shown in Fig. 2D and quantification in Fig. 2E, Y645A, W667A, and Y669A mutant EGFP-BAHD1 failed to co-localize with H3K27me3 compared with the wild type protein, thus supporting the functional importance of the aromatic cage for heterochromatin targeting by BAHD1 *in vivo*.

In sum, combining peptide array screen, quantitative binding, hydrogen exchange MS, and cellular co-localization studies, we established that BAHD1 BAH domain is an H3K27me3-specific reader that discriminates against H3K9me3. H3K27me3 often marks facultative heterochromatin with important functional implications in gene regulation, cell differentiation and development (Gaydos, Wang et al. 2014). By contrast, histone H3K9me3 represents a hallmark for constitutive heterochromatin and can be recognized by effector proteins such as HP1 to maintain structurally condensed chromatin conformation. Our quantitative ITC assays revealed that the binding affinity of BAH_BAHD1_ to H3K27me3 (*K*_D_ = 15.9 μmol/L) is more than two orders of magnitude stronger than H3K9me3 (*K*_D_ = 2.6 mmol/L). This recognition preference suggests that distal sequences flanking the K9/K27 consensus “ARKS” motif contribute to H3K27me3 recognition. In support, an optimal binding between H3K27me3 and BAH_BAHD1_ is achieved in a long frame of 15–42 but not in shorter segments of 19–34 or 21–32. Exact molecular basis underlying H3 distal sequence recognition calls for complex structure determination in the future. Utilizing hydrogen exchange MS, we were able to map key segments within BAH_BAHD1_ that are responsible for H3K27me3 binding. Notably, spatial arrangement of these key segments in modelled BAH_BAHD1_ structure underscored the role of an aromatic cage consisting of Y645, W667, and Y669 for H3K27me3 readout, a mechanism conserved in many other methyllysine readers (Patel and Wang 2013). Subsequent ITC titration and immunofluorescence studies comparing wild type and mutant BAH_BAHD1_ further confirmed the importance of the aromatic cage in histone H3K27me3 readout both *in vitro* and *in vivo.*

Previously reported H3K27me3 readers include EED WD40 repeats of the PRC2 complex (Margueron, Justin et al. 2009) and Pc family chromodomain of the PRC1 complex (Kaustov, Ouyang et al. 2011). Here we characterized human BAHD1 BAH domain as a third class of histone H3K27me3 reader that functions to facilitate BAHD1 heterochromatin targeting and subsequent gene silencing. Moreover, our work revealed that the binding of BAH_BAHD1_ to H3K27me3 was markedly disrupted by adjacent H3S28 phosphorylation—a hallmark of the transcriptional response to stress signaling (Sawicka, Hartl et al. 2014). BAHD1 is overexpressed in peripheral blood mononuclear cells and pancreas, and excels critical function to maintain a repressive state of interferon-stimulated genes and insulin-like growth factors (Bierne, Tham et al. 2009, Lebreton, Lakisic et al. 2011). Given the critical role of H3S28ph in signal-induced transcription, the observed “binary switch” between H3K27me3 and H3S28ph may serve as an important mechanism to derepress BAHD1-mediated gene silencing.

## Electronic supplementary material

Supplementary material 1 (PDF 1035 kb)
