# Supplementary material for: The BAH domain of BAHD1 is a histone H3K27me3 reader
Source: Protein Cell. 2016 Feb 5;7(3):222–6. doi: 10.1007/s13238-016-0243-z (PMC4791424; doi:10.1007/s13238-016-0243-z)
Supplement: Supplementary file 1 — Supplementary material 1 (PDF 1035 kb) [file 13238_2016_243_MOESM1_ESM.pdf]

Supplementary Information

The BAH domain of BAHD1 is a histone H3K27me3 reader

Dan Zhao[1, 2], Xiaojie Zhang[2], Haipeng Guan[2], Xiaozhe Xiong[2], Xiaomeng Shi[3], Haiteng Deng[3], Haitao Li[2, 4]

[1]*College of Life Sciences, Peking University, Beijing 100871, P.R. China*

[2]*MOE Key Laboratory of Protein Sciences, Center for Structural Biology, Department of Basic Medical Sciences, School of Medicine, Tsinghua University, Beijing 100084, P.R. China*

[3]*Center for Biomedical Analysis, School of Medicine, Tsinghua University, Beijing 100084, China*

[4]*Collaborative Innovation Center for Biotherapy, West China Hospital, Sichuan University, Chengdu 610041, P.R. China*

To whom correspondence should be addressed:
Haitao Li
Medical Science Building C228
Tsinghua University
Beijing 100084, P.R.China
Tel: (+) 86-10-62771392
E-mail: lht@tsinghua.edu.cn

Running title: H3K27me3 readout by BAHD1

Keywords: Histone modification, BAH domain, H3K27 trimethylation, Heterochromatin formation, Hydrogen exchange MS, Epigenetic regulation

Materials and Methods

Materials

The full-length gene of human BAHD1 (residues 1-780) was amplified from HEK293 cDNA library by PCR and verified by sequencing. Histone peptides bearing different modifications were synthesized by SciLight Biotechnology, LLC. Anti-H3K27me3 (PTM-622) antibody was obtained from PTM-BioLabs and the anti-GST antibody (M20007) was obtained from Abmart.

Protein expression and purification

The BAH domain encompassing residues 589-780 of human BAHD1 was cloned in to a pSUMOH10 vector (an in house modified vector based on pET28b) containing an N-terminal 10xHis-SUMO tag. All BAH_{BAHD1} mutants were generated using QuikChange (Stratagene) method and verified by gene sequencing. The recombinant $BAHD1_{589\text{-}780}$ was overexpressed in E.coli BL21 (DE3). After overnight induction by 0.2 mM isopropyl β-D-thiogalactoside (IPTG) at 16 °C in TB medium, cells were harvested and suspended in buffer: 20 mM Tris, pH 8.5, 0.5 M NaCl, 5% glycerol. After cell lysis and centrifugation, the recombined protein was purified to homogeneity over HisTrap, and the10xHis-SUMO tag was cleaved by ULP1 overnight at 4°C then removed by reloaded onto the HisTrap column. The free BAH_{BAHD1} protein was collected via size-exclusion chromatography on Superdex G75 column (GE Healthcare) in elution buffer: 20 mM Tris, pH 8.5, 0.5 M $(NH)_2SO_4$, 5% glycerol. The purification procedures for BAH_{BAHD1} mutants were essentially the same as the wild type protein.

For peptide array screening, the GST-tagged BAH_{BAHD1} was cloned in pGEX-6P-1 and overexpressed in E.coli BL21 (DE3). After overnight induction by 0.2 mM isopropyl β-D-thiogalactoside (IPTG) at 16 °C in TB medium, cells were harvested and suspended in buffer: 20 mM Tris, pH 8.5, 0.5 M NaCl, 5% glycerol. After cell lysis and centrifugation, the recombined proteins were loaded onto a GST affinity column. Bound proteins were eluted with 20 mM glutathione and purified via size-exclusion chromatography on a Hiload 16/60 Superdex 75 column (GE Healthcare) in elution buffer: 20 mM Tris, pH 8.5, 0.5 M $(NH)_2SO_4$, 5% glycerol.

Modified histone peptide array

The modified histone peptide array was preformed essentially as indicated by the manufacturer (Active Motif, Cat 13001&13005). Briefly, the arrays were blocked with 5% skim milk in TTBS buffer (20 mM Tris, pH 7.5, 150 mM NaCl, 0.05% Tween-20 and 1 mM PMSF) overnight and then incubated with 15 μM GST-tagged protein in binding buffer (20 mM Tris, pH 8.5, 300 mM NaCl) for 5 h at 4 °C. The arrays were washed three times for 10 minutes each using TTBS buffer. Added anti-GST antibody (1: 2000 dilution; Cat: CW0085) for 8 at 4 °C, then washed three times for 10 minutes each. Incubated with second antibody (anti-rabbit, 1:4000 dilution) for 1h at RT and then wash three times before ECL reaction.

Fluorescence-based thermal shift assay (TSA)

The TSA was performed with a CFX96 real-time PCR instrument (Bio-Rad). A typical TSA solution was composed of 1 mg/mL BAH_{BAHD1} protein, Sypro Orange (Invitrogen) and 5 different kinds of salts (500 mM sodium chloride, sodium formate, sodium acetate trihydrate, sodium nitrate, ammonium sulfate) in buffer containing 20 mM Tris, pH 8.5, 5% glycerol. During TSA assays, all samples were heated from 15°C to 75°C at a rate of 0.5°C per minute. Protein denaturation was monitored by increased fluorescence signal of Sypro Orange, which captures exposed hydrophobic residues during thermal unfolding. The recorded curves were analyzed by the software CFX-Manager (Bio-Rad). The temperature corresponding to the inflection point was defined as the melting temperature, Tm.

Isothermal titration calorimetry (ITC) measurements

All calorimetric experiments of the wild type or mutant BAH_{BAHD1} were conducted at 15 °C using a MicroCal iTC200 instrument (GE Healthcare). The BAH_{BAHD1} samples were dialyzed in the following buffer: 20 mM Tris, pH 8.5, 500 mM $(NH)_2SO_4$ and 5% glycerol. Protein concentration was determined absorbance spectroscopy at 280 nm. Peptides were quantified by weighing on a large scale and then aliquoted and freeze-dried for individual use. Acquired calorimetric titration curves were analyzed using Origin 7.0 (OriginLab) using the "One Set of Binding Sites" fitting model. Detailed thermodynamic parameters of each titration were summarized in Supplementary Table S1.

Cell culture and transfection

Human HeLa cells (ATCC) were maintained in Dulbecco’s modified Eagle’s medium (Gibco) containing 10% fetal bovine serum (Gibco) and supplemented with 100 U/mL penicillin, and 100 μg/mL streptomycin. Plasmid transfection was performed using lipofectamine 2000 (Invitrogen) according to the manufacturer’s instructions.

Immunofluorescence staining

HeLa cells were fixed in 4% (w/v) formaldehyde in PBS for 10 min, permeabilized in 0.2% (v/v) Triton X-100 in PBS for 5 min. Then washed cells twice using PBS. After incubation with primary antibody against H3K27me3 (1:200, ptm-biolab, PTM-622) in 3% BSA overnight at 4°C, cells were washed and probed with Alexa fluor-conjugated secondary antibodies in 3% BSA for 1 hr. Cells on the slide were then washed, covered with mounting medium (DAPI Fluoromount-G, Southern Biotech, 0100-20), and observed under Zeiss Laser Scanning Confocal Microscopy (LSM710). Immunofluorescent images were taken as Z-stacks with a DeltaVision image restoration microscope system. Exposure times and settings for deconvolution were constant for all samples to be compared within any given experiment. The image quantification was performed by Imaris (Bitplane).

Deuterium labeling and mass spectra analysis

To prepare for labeling, all protein solutions were brought to a concentration of 180 μM. For experiments with BAH_{BAHD1} protein alone, 2 μl of 180 μM protein was diluted with 18 μl of 20 mM Tris, 500 mM $(NH)_2SO_4$, pH 8.5. For experiments with BAH_{BAHD1} bound to $H3_{15-42}K27me3$ peptide, 2 μl of 180 μM protein was diluted with 18 μl of 20 mM Tris, 500 mM $(NH)_2SO_4$, pH 8.5. To initiate deuterium labeling, 5 μl of each 180 μM protein solution was diluted with 45 μl of labeling buffer (20 mM Tris, 500 mM $(NH)_2SO_4$, 99% D2O, pH 8.5) at room temperature. At 10 minutes, the labeling reaction was quenched by adding 50 μl of ice-cold quench buffer (fomic acid in water solution at pH 1.3, 100% H2O), then immediately put the sample tube on ice. 5μl 1μM pepsin solution was added for digestion. At 5 minutes, the sample was placed into Waters nanoACQUITY UPLC autosampler for injection.

5 μl sample was loaded onto a peptide trap (nanoACQUITY UPLC 20-VM trap Symmetry C18 180umx20mm, Waters). A nanoACQUITY UPLC 1.7um BEH 30 C18 75umx100mm column was used to separate digested peptides. A 10% to 40% gradient of acetonitrile over 20 min at a flow rate of 0.35 μl min−1 was used to separate peptides. Both chromatographic mobile phases contained 0.05% (v/v) formic acid. Mass analysis was conducted with a SYNAPT G2-Si mass spectrometer (Waters) equipped with standard ESI source and lockmass correction. Glu-fibrinopeptide was used to maintain the calibration at 3–5 p.p.m. throughout analysis. The mass spectrometer settings were: ESI+ mode; capillary, 3,200 V; cone, 40 V; source temperature, 80 °C; mass acquisition range, 400–1,800 m/z; scan rate, 0.6 scans per s; instrument always collecting data in MSE mode. Peptic peptides were identified using MSE and IdentityE software within ProteinLynx Global Server 3.0.2 (PLGS) (Waters). The deuterium exchange levels were determined by subtracting the centroid mass of undeuterated peptide from the centroid mass of deuterated peptide using HX-Express (Weis, Engen et al. 2006).

Supplemental Table S1

Summary of thermodynamic parameters from ITC

Protein	Peptides	ΔH (kcal/mol)	ΔS (cal/mol/deg)	Kd (μM)	N
$BAH_{589-780}$	$H3_{15-42}$un	N.D.			
	$H3_{15-42}$K27me1	-25.7	-77.1	2045	1.0
	$H3_{15-42}$K27me2	-14.8	-33.4	116.3	1.1
	$H3_{15-42}$K27me3	-11.5	-17.9	15.9	1.0
	$H3_{15-42}$K27me3S28ph	-13.4	-31.9	680.3	1.0
	$H3_{21-32}$K27me3	-8.3	-10.9	123.8	1.1
	$H3_{19-34}$K27me3	-13.7	-28.4	61.3	0.9
	$H3_{1-15}$K9me3	-37.9	-120	2591	1.0
	$H3_{1-25}$K14ac	N.D.			
	$H3_{1-10}$R8me2s	N.D.			
	$H3_{28-41}$K36me3	N.D.			
Y645A	$H3_{15-42}$K27me3	-6.1	-7.4	990.0	1.0
W667A	$H3_{15-42}$K27me3	N.D.			
Y669A	$H3_{15-42}$K27me3	N.D.			

$H3_{15-42}$un: APRKQLATKAARKSAPATGGVKKPHRYR

$H3_{15-42}$K27me1: APRKQLATKAARK(me1)SAPATGGVKKPHRYR

$H3_{15-42}$K27me2: APRKQLATKAARK(me2)SAPATGGVKKPHRYR

$H3_{15-42}$K27me3: APRKQLATKAARK(me3)SAPATGGVKKPHRYR

$H3_{15-42}$K27me3S28ph: APRKQLATKAARK(me3)S(ph)APATGGVKKPHRYR

$H3_{21-32}$K27me3: ATKAARK(me3)SAPAT

$H3_{19-34}$K27me3: QLATKAARK(me3)SAPATGG

$H3_{1-15}$K9me3: ARTKQTARK(me3)STGGKA

$H3_{1-15}$K14ac: ARTKQTARKSTGGK(ac)A-NH_2

$H3_{1-10}$R8me2s: NH_2-ARTKQTAR(me2s)KS-COOH

$H3_{28-41}$K36me3: Ac-SAPSTGGVK(me3)KPHRY-NH_2

N.D. not detected

Supplemental Table S2

Pearson's coefficient in co-localized volume of WT and mutants with H3K27me3

WT	Y645A	W667A	Y669A
0.6369	0.2769	0.2701	0.4849
0.55	0.2962	0.2537	0.525
0.561	0.3329	0.271	0.3619
0.558	0.2816	0.2559	0.5284
0.6896	0.2532	0.2505	0.5164
0.5288	0.2251	0.2185	0.4145
0.606	0.3237	0.3051	0.435
0.6402	0.3011	0.2657	0.5258
0.5744	0.3189	0.3005	0.4097
0.5499	0.3788	0.238	0.4001
0.6724	0.2971	0.2699	0.3957
0.6892	0.3017	0.2194	0.3557

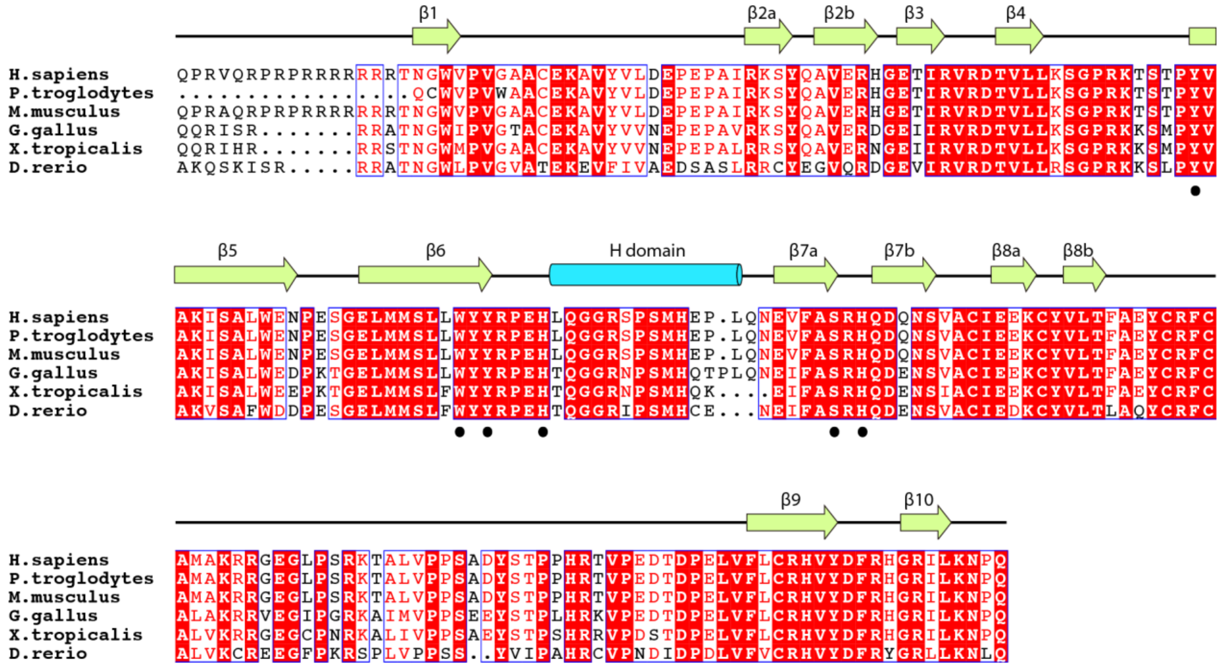

Supplementary Figure S1. Sequence alignment of BAHD1 orthologs.

Amino acid sequences of BAH_{BAHD1} domain from Homo sapiens, Pan troglodytes, Mus musculus, Gallus gallus, Xenopus tropicalis, Danio rerio are aligned with Clustal Omega (Sievers, Wilm et al. 2011) and graphically displayed with ESPript (Gouet, Courcelle et al. 1999).The conserved residues are highlighted in red. The secondary structure is drawn based on the structural model of BAHD1. The potential methyl-lysine binding residues are designated by black circle.

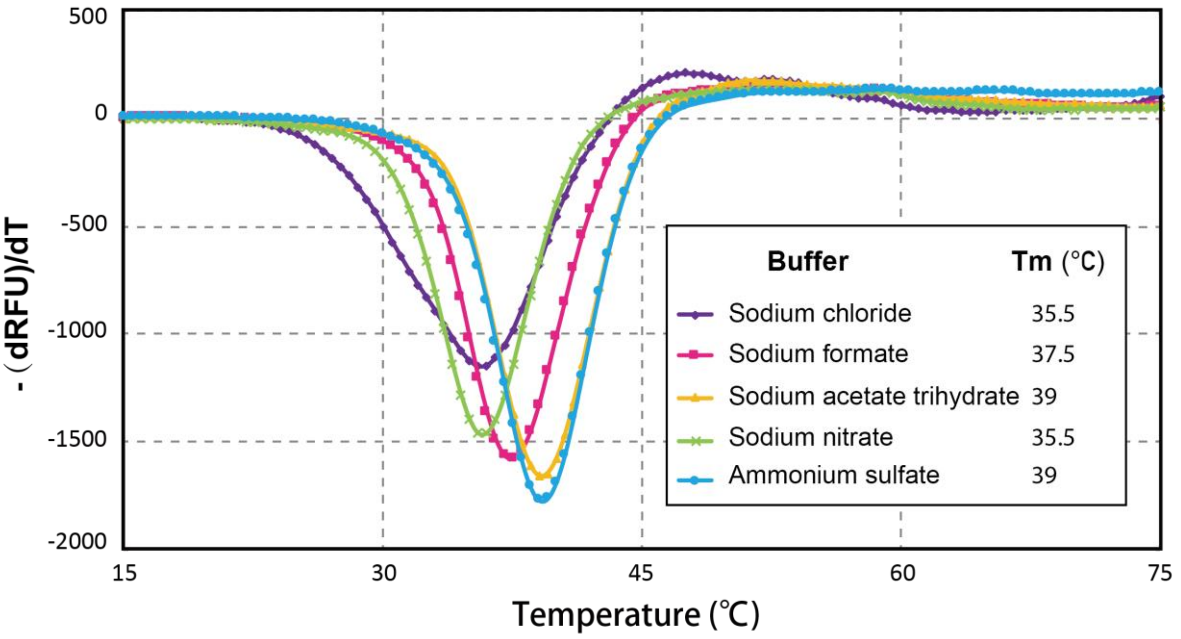

Supplementary Figure S2. Buffer screening for BAH $_{\text{BAHD1}}$ domain by Thermofluor shift assay (TSA).

All assays were performed in the solutions containing 500mM each salt (show above) in the presence of same buffer (20mM Tris, pH 8.5, 5% glycerol).

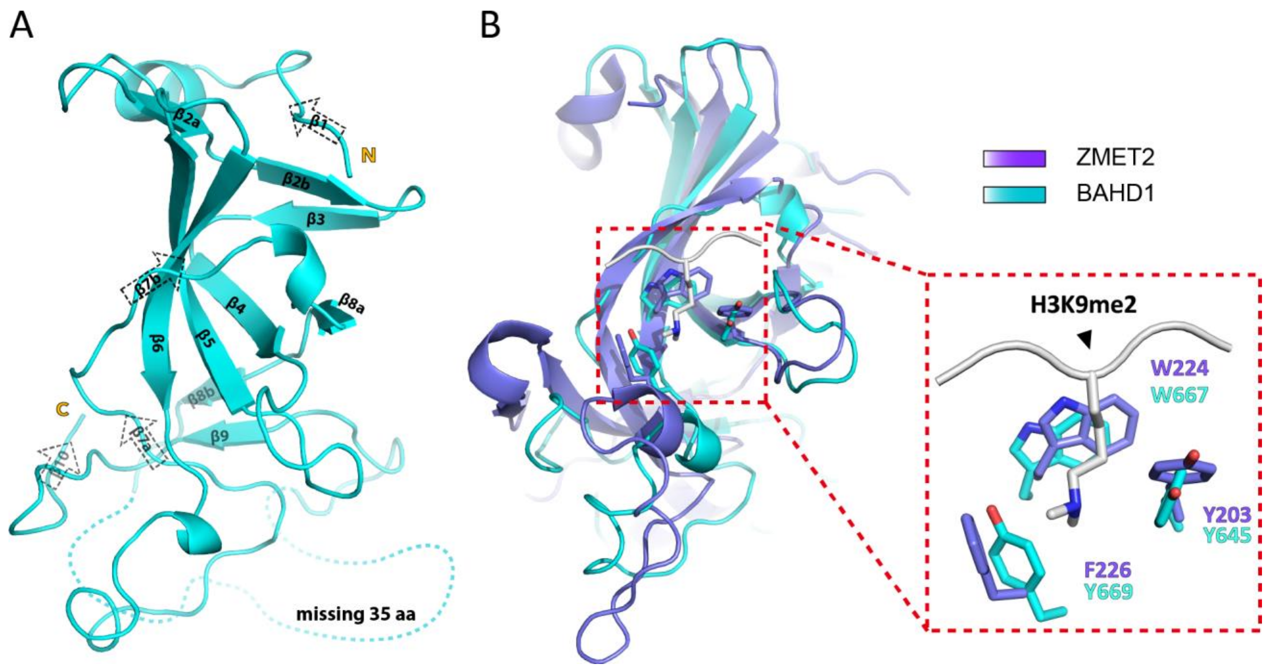

Supplementary Figure S3. Structural model of BAH_{BAHD1} and superimposition with BAH_{mORC1}.

(A) Overall structural model of BAH_{BAHD1} domain which was predicted by the online server Phyre2 (http://www.sbg.bio.ic.ac.uk/phyre2/html/) (Kelley, Mezulis et al. 2015). BAHD1 BAH domain is shown as cyan ribbon. The tentative β strands elements (reported in other BAH domain structures) that are modelled as loops are denoted by dashed arrows. (B) Structural superposition of BAH domains from human BAHD1 and plant ZMET2. The BAH domain of ZMET2 (PDB: 4FT4) is shown as purple ribbon with key residues of K9me2 pocket depicted as stick. The predicted BAH domain of BAHD1 is shown as cyan ribbon with conserved methyl-lysine recognition aromatic residues shown as stick. Dashed box, a close-up view of the superimposed aromatic cage with bound H3K9me2.

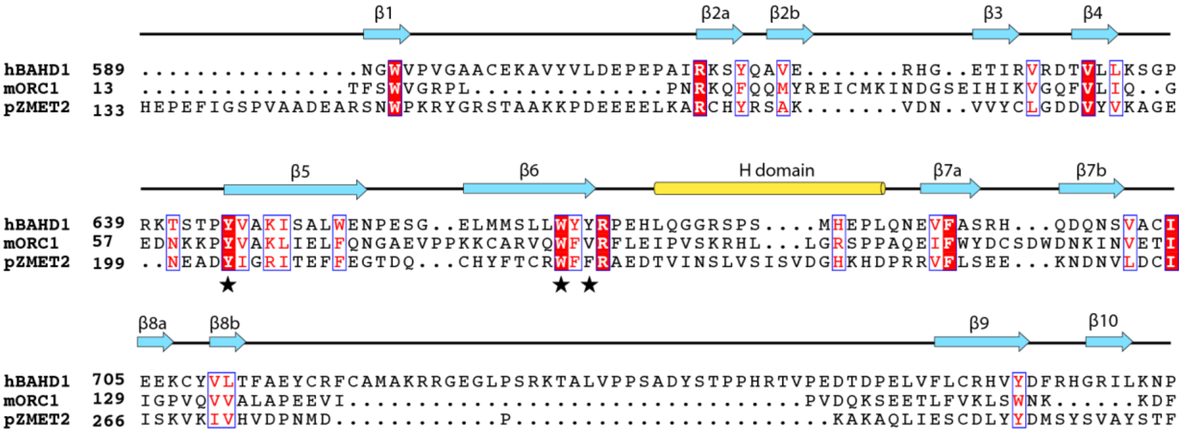

Supplementary Figure S4. Structure-based sequence alignment of BAH domain of human BAHD1, mouse ORC1, and plant ZMET2.

Conserved residues are shaded in red; the residues involved in “methyl-lysine” binding are highlighted with black stars. (Gouet, Courcelle et al. 1999)

Reference

Gouet, P., E. Courcelle, D. I. Stuart and F. Metoz (1999). "ESPript: analysis of multiple sequence alignments in PostScript." Bioinformatics **15**(4): 305-308.

Kelley, L. A., S. Mezulis, C. M. Yates, M. N. Wass and M. J. Sternberg (2015). "The Phyre2 web portal for protein modeling, prediction and analysis." Nat Protoc **10**(6): 845-858.

Sievers, F., A. Wilm, D. Dineen, T. J. Gibson, K. Karplus, W. Li, R. Lopez, H. McWilliam, M. Remmert, J. Soding, J. D. Thompson and D. G. Higgins (2011). "Fast, scalable generation of high-quality protein multiple sequence alignments using Clustal Omega." Mol Syst Biol **7**: 539.

Weis, D. D., J. R. Engen and I. J. Kass (2006). "Semi-automated data processing of hydrogen exchange mass spectra using HX-Express." J Am Soc Mass Spectrom **17**(12): 1700-1703.
